# Human pancreatic islet-derived extracellular vesicles modulate insulin expression in 3D-differentiating iPSC clusters

**DOI:** 10.1371/journal.pone.0187665

**Published:** 2017-11-08

**Authors:** Diana Ribeiro, Eva-Marie Andersson, Nikki Heath, Anette Persson-kry, Richard Collins, Ryan Hicks, Niek Dekker, Anna Forslöw

**Affiliations:** 1 Discovery Sciences, Innovative Medicines and Early Development Biotech Unit, AstraZeneca, Gothenburg, Sweden; 2 Department of Biology and Bioengineering, Chalmers University of Technology, Gothenburg, Sweden; 3 Cardiovascular and Metabolic Diseases, Innovative Medicines and Early Development Biotech Unit, AstraZeneca, Gothenburg, Sweden; 4 Discovery Sciences, Innovative Medicines and Early Development Biotech Unit, AstraZeneca, Alderley Park, Macclesfield, United Kingdom; 5 Faculty of Life Sciences, University of Manchester, Manchester, United Kingdom; University of Pécs Medical School, HUNGARY

## Abstract

It has been suggested that extracellular vesicles (EVs) can mediate crosstalk between hormones and metabolites within pancreatic tissue. However, the possible effect of pancreatic EVs on stem cell differentiation into pancreatic lineages remains unknown. Herein, human islet-derived EVs (h-Islet-EVs) were isolated, characterized and subsequently added to human induced pluripotent stem cell (iPSC) clusters during pancreatic differentiation. The h-islet-EVs had a mean size of 117±7 nm and showed positive expression of CD63 and CD81 EV markers as measured by ELISA. The presence of key pancreatic transcription factor mRNA, such as NGN3, MAFA and PDX1, and pancreatic hormone proteins such as C-peptide and glucagon, were confirmed in h-Islet-EVs. iPSC clusters were differentiated in suspension and at the end stages of the differentiation protocol, the mRNA expression of the main pancreatic transcription factors and pancreatic hormones was increased. H-Islet-EVs were supplemented to the iPSC clusters in the later stages of differentiation. It was observed that h-Islet-EVs were able to up-regulate the intracellular levels of C-peptide in iPSC clusters in a concentration-dependent manner. The effect of h-Islet-EVs on the differentiation of iPSC clusters cultured in 3D-collagen hydrogels was also assessed. Although increased mRNA expression for pancreatic markers was observed when culturing the iPSC clusters in 3D-collagen hydrogels, delivery of EVs did not affect the insulin or C-peptide intracellular content.

Our results provide new information on the role of h-Islet-EVs in the regulation of insulin expression in differentiating iPSC clusters, and are highly relevant for pancreatic tissue engineering applications.

## Introduction

Diabetes mellitus (DM) is a chronic metabolic disorder characterized by hormonal dysregulation in insulin-producing pancreatic β-cells [1, 2]. Disease incidence, particularly for type 2 DM, is increasing worldwide, and has an impact on the affected individual as well as the health care systems [3, 4]. Disease modelling and development of clinical treatment options are impaired by limited availability and functionality of isolated pancreatic islets from donors [5, 6].

The advancement of pluripotent stem cell technology has led to a cascade of technological innovations in stem cell-based differentiation [7], with the potential to generate alternative sources of islet-like cells [8]. Common differentiation protocols use complex and sequential cocktails of growth factors and/or small molecules to direct endocrine differentiation of stem cells [9–11], and more recently this has been used in combination with stem cell clusters cultured in suspension [12–15]. These clusters resemble islet 3D-architecture and show increased functional insulin release properties in-vitro [16–18]. Earlier literature supports the use of 3D-culture, to preserve and/or increase in-vitro pancreatic islet viability and insulin secretion [19, 20]. Of particular interest are 3D-scaffolds containing extracellular matrix (ECM) motifs found within the pancreatic ECM, such as collagen [21, 22].

Despite increased efforts in the development of complex in-vitro 3D-stem cell culture systems, previously mentioned differentiation protocols may not provide the dosage, temporal and synergistic signals that constitute the pancreatic islet in-vivo niche.

It has been shown that protein, lipid and RNA packaged into and released from cells in extracellular vesicles (EVs) can modify/reprogramme the phenotype of recipient cells. [23]. EVs are circulating, cell-derived membrane enclosed vesicles involved in the communication between cells [24]. Several types of EVs have been described in the literature including plasma membrane derived microvesicles and exosomes which originate from within the endosomal system [25–27]. Recent literature supports a role for EVs in metabolic-associated disturbance [28], in particular β-cell insulin resistance and overall potential to affect development of DM [29]. In fact, microvesicles isolated from pancreatic β-cell lines were found to transfer microRNAs to neighboring β-cells. Furthermore, β-cell microvesicular cargo content was affected by exposure to pathophysiological conditions associated with DM [30]. Another notable study showed that biologically active EVs released from human pancreatic islets were able to shuttle specific microRNAs to human islet endothelial cells, inducing *INS* mRNA expression, offering protection from apoptosis and enhancement of angiogenesis [31]. However, the effect of pancreatic islet-derived EVs on stem cell differentiation and pancreatic commitment remains unknown.

Here, we test the hypothesis that EVs isolated from human pancreatic islets can deliver RNA and/or proteins to differentiating cells, and influence the outcome of the differentiation towards insulin-producing cells. EVs were isolated from human pancreatic islets (h-islets) and their concentration, size and specific pancreatic cargo was characterized. Next, the effect of the addition of h-islet-EVs to differentiating pancreatic iPSC clusters was assessed in both suspension cultures and in hydrogels composed of collagen type 1.

## Materials and methods

### Materials and media

Materials and media used throughout this work can be found in Materials and methods A in S1 File.

### Human pancreatic islets

Human primary islets were purchased from Prodo Laboratories Inc., who provide islets isolated from donor pancreases obtained from deceased individuals with research consent from Organ Procurement Organizations (OPOs). The use and storage of human islets was performed in compliance with the Declaration of Helsinki, ICH/Good Clinical Practice, and AstraZeneca code of conduct. Samples were anonymized prior to receiving them from Prodo Laboratories Inc. H-Islets were transported in PIM (S) ® and changed to fresh PIM® for an overnight recovery. Donor information can be found in Table A in S1 File.

### EV isolation

PIM® media supplemented with human serum was used for islet culture (PIM® complete). PIM® media itself does not contain any other animal-derived product. PIM® complete media was used to extract EVs corresponding to the human serum fraction, which were used as control EVs (h-Ctr-EVs). PIM® media was purchased routinely at each islet preparation, and different batches were used. Human pancreatic islet-derived EVs were isolated from pancreatic islet conditioned media after one overnight culture in PIM® complete media. Conditioned media was centrifuged at 200 g for 10 minutes, followed by 2000 g for 10 minutes and a final centrifugation of 4000 g for 60 minutes. The supernatant was filtered with a 0.45 μm filter and incubated with ExoQuick-TC^TM^ reagent following manufacturer instructions. EVs were characterized for concentration, surface expression markers, size distribution using NTA, and visualised using TEM.

### EV quantification

EVs were quantified in terms of total protein content and particle concentration. EV total protein content was measured using a Pierce™ BCA Protein Assay Kit. Particle concentration was measured using the EXOCET exosome Quantification assay kit and Nanoparticle tracking analysis (NTA). NTA was carried out using the LM10 Nanosight (Malvern) equipped with a sample chamber and a 405 nm laser. EVs were diluted in PBS to fit in the resolution window of measurement suggested by the instrument manufacturer. Samples were injected into the sample chamber using a 1 mL syringe until the chamber was filled and sample was starting to exit the system chamber outlet. Three separate fields of view were captured for each sample for 30 second measurements at room temperature. A script was used which allowed the sample to be advanced manually between each measurement. The Nanosight NTA 3.1 software was used to capture and analyze the particles. Camera levels, screen gain and detection thresholds were kept consistent between replicates of samples but not between different sample conditions. The expression of tetraspanins was measured with Exo-ELISA for CD63, CD9 and CD81 following manufacturer´s instructions.

### Transmission electron microscopy

Negative stain transmission electron microscopy was used to image the EVs. EVs were re-suspended in TBS and frozen at -80°C. TBS was found to be a more desirable buffer for analysis of EVs via TEM (unpublished data). Carbon film on gold 300 square mesh grids (EM resolutions) that had been subjected to glow discharge for 2 minutes at 25 mA, were applied to 10 μl of thawed EVs and left to adsorb for 1 minute. Excess liquid was blotted using Whatman blotting paper and a grid was applied to 10 μL 2% uranyl acetate (Agar Scientific) for 1 minute. Excess liquid was blotted and grids were stored for use. Grids were imaged on a Tecnai 12 BioTwin coupled to a GATAN Orius 2K CCD camera transmission electron microscope operating at 120 KV.

### EV cargo characterization

EVs were assessed for the presence of RNA and protein of several pancreatic markers and hormones. RNA was extracted based on a combination of phenol-based method and a final purification step using the RNeasy® Mini Kit. Briefly, EV pellets were lysed in TRIzol® and frozen. For each 700 μL of TRIzol® solution 90 μL of chloroform was added, and the mix was vortexed for 15 seconds and incubated for 2 minutes at room temperature. The mix was centrifuged at 14000 g for 15 minutes at 4°C. From this centrifugation step resulted a 3 phase mix, in which was an upper colourless aqueous phase containing RNA. This phase was transferred to a clean Eppendorf tube, to which 2 times volume of absolute ethanol was added. This solution was transferred to the spin columns of the RNeasy® Mini Kit, following the manufacturer´s instructions. The protocol followed for quantification of gene expression is detailed in Materials and methods B in S1 File. The pancreatic hormone quantification is described below in the ELISA section. 25–50 μg of EVs were run on the ELISAs.

### Pancreatic differentiation in suspension

Undifferentiated iPSC clusters were generated according to Materials and methods C in S1 File. Suspension differentiation was carried out in Corning® 125 mL baffled Erlenmeyer flasks, using 40 mL media per flask. The differentiation protocol used was adapted from Russ et al. [15] and the details described in Material and methods D in S1 File.

### EV supplementation

iPSC clusters, at day 11 of the differentiation protocol, were transferred to 96 well spheroid plates and incubated with various concentrations of h-Islet-EVs (0, 100, 200 and 300 μg EV_Protein_ /mL) at every media change. iPSC clusters were allowed to sediment for 1 to 3 minutes and media was removed without disturbing the cluster pellet. The cluster pellet was resuspended with fresh media+EVs, at a concentration of 1.5% (V_iPSC cluster_/V_media_). 100 μL of this solution was added to each 96 well.

### 3D-culture in collagen hydrogels

Cell clusters were mixed with the freshly prepared collagen solutions (Material and methods C in S1 File). A cell cluster suspension was added to the gel mix at 10% (V_iPSC cluster_/V_gel_) concentration. Clusters were pre-differentiated in suspension prior to encapsulation in 3D-culture in hydrogels, and differentiation in 3D-hydrogels was carried out following the same differentiation media and media change timing. EVs were supplemented by addition to the differentiation media at 200 μg of EV_protein_/mL.

### Gene expression analysis

At different culturing time points, samples were taken from the cell cluster suspensions or 3D cultures for RNA analysis. Gene expression procedures are detailed in Materials and methods B in S1 File. Briefly, The RNA extraction of cell suspension clusters or encapsulated collagen-clusters was performed using the RNeasy® Mini Kit. RNA was converted to cDNA using the using a High-Capacity cDNA Reverse Transcription Kit. Quantitative real-time PCR (qPCR) was performed using TaqMan® reagents. Primer information is described in Table B in S1 File.

### Protein quantification by ELISAs

The pancreatic hormones insulin, C-peptide and glucagon, were quantified using the Mercodia Insulin ELISA, Mercodia ultrasensitive C-peptide ELISA and Mercodia Glucagon ELISA. Cell samples were washed in PBS and lysed in H_2_O. Data was normalized against the total DNA content using the Quant-iT™ PicoGreen ® dsDNA.

### Apoptosis

Apoptosis was measured using the Luminescent Caspase Assay, following manufacturer´s instructions.

### Statistical analysis

A minimum of three independent experiments were performed, with a minimum of two technical replicates per condition and assay. Data was statistically analysed using GraphPad Prism software (version 6.01; GraphPad Software Inc.). Automatic correction for outliers was performed using the ROUT method (Q = 1%). The results were analysed for normal distribution. Parametric or non-parametric methods were used to assess variance in the data depending of the data normality. Statistical significance of calculated P-values is defined in Table C in S1 File, P-values of 0.05 or less were considered statistically significant in the analysis of the results. Boxplots graphs were used to display the distribution of data.

## Results and discussion

### h-Islet-EV concentration and size characterization

**Fig 1:**
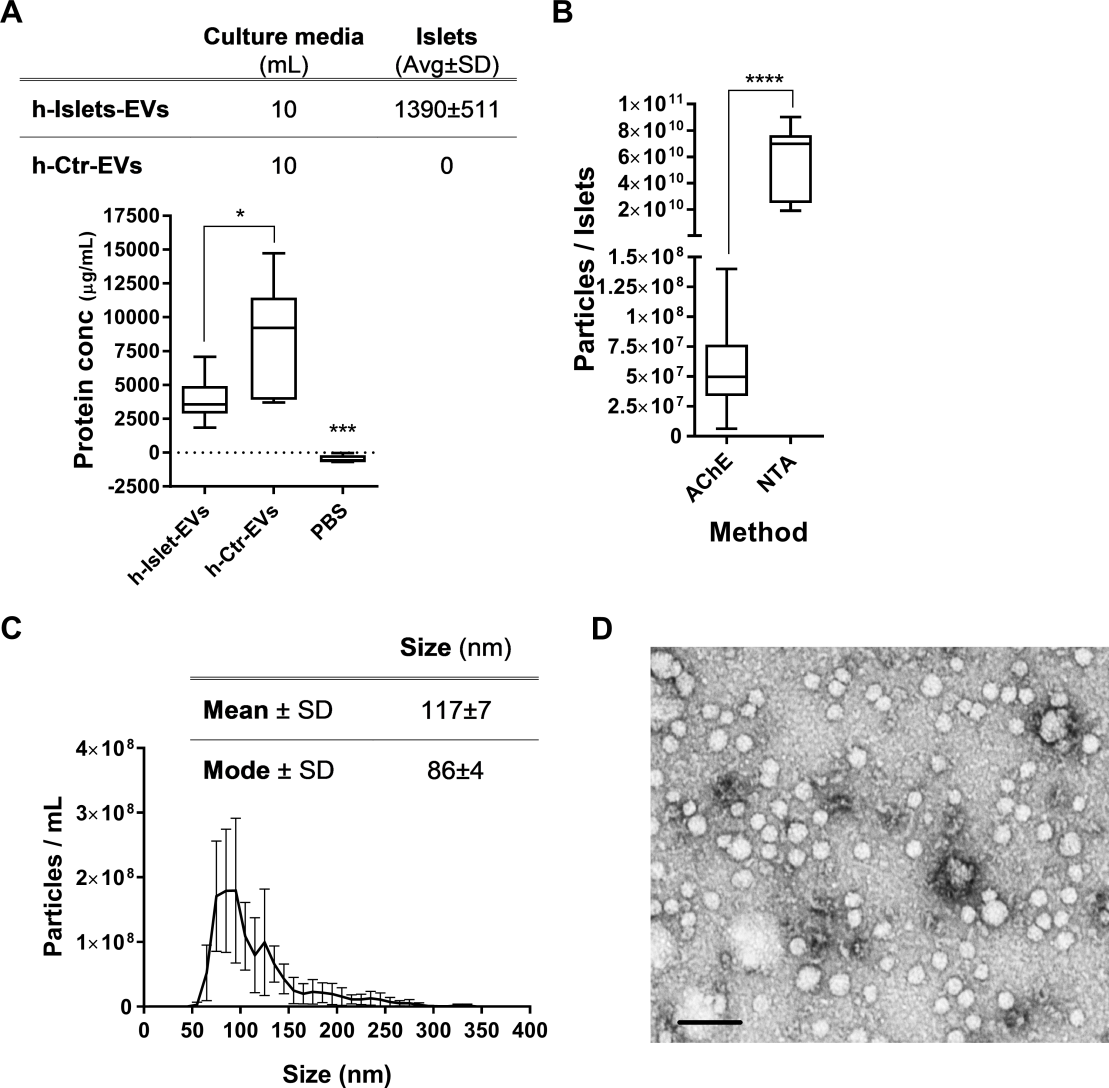
Characterisation of EVs. A) h-Islet-EVs (N = 11) and h-Ctr-EVs (N = 5) protein concentration per 10 mL of isolation media. For each 10 mL of media containing h-Islets, an average of 1390±511 h-Islets were present. No islets were cultured in the media for the control condition. B) Particle concentration was quantified using the AChE activity assay (N = 11) or nanoparticle tracking analysis (N = 3). C) Size distribution over frequency of events (N = 3). Descriptive statistics listed in table above the graph. D) Representative TEM image. Scale bar = 100 nm. * represent statistical significance, and the corresponding p-value in Table C in S1 File.

Human islets (h-islets) are a delicate cell population, limited in availability but of great interest in diabetes research. The potential to use islet-derived conditioned media for the development of functional assays adds great value to the field. In this study, EVs were isolated from the conditioned media of non-diabetic h-islet donors with an average age of 41±13 years and an average BMI of 27±4. Human islets are sensitive to isolation and insulin regulation becomes unbalanced in in-vitro culture [5]. To maintain the h-islet phenotype post isolation and transportation, h-islets were cultured in medium supplemented with human serum (PIM complete). After an overnight incubation, media conditioned by the h-islets was harvested and EVs were isolated. PIM complete media is supplemented with human serum which is documented to contain EVs [32, 33], and thereby control EV samples (h-Ctr-EVs) were also isolated from media that had not been exposed to h-islets. H-Islet-EVs had a significantly lower protein concentration compared to h-Ctr-EVs (Fig 1A). The same pattern was observed when analyzing the ratio of particle number per μg of protein for both EV fractions (Figure A in S1 File). One can speculate that human islets may have taken up and processed h-Ctr-EVs present in the PIM conditioned media, and thereby h-islet-EVs subsequently released were not visible as a cumulative release effect. The protein content of h-Islet-EVs showed less sample variability, as compared to Ctr-EVs, indicated by the interquartile distribution of the box-plot.

The concentration of h-Islet-EVs was further characterized by measuring Acetyl-CoA Acetylcholinesterase (AChE) activity, known to be enriched within EVs [34], and by nanoparticle tracking analysis (NTA) (Fig 1B). The results showed significant variance depending on the method used. The NTA data showed a higher concentration of h-islet-EVs as compared to the AChE method, which may be explained by the presence of AChE in only a sub-population of EVs. The EV size distribution was analyzed using NTA and the average h-Islet-EV size was 117±7 nm and mode size of 86±4 nm (Fig 1C). EVs have previously been isolated from media conditioned by human islets by ultracentrifugation [31], the EVs in this study had a mean size of 236±5 compared to 117±7 nm. The difference in size may be due to different methods of isolation, although the size range observed in both studies is in agreement with current literature about the EVs [25, 27]. Regarding the isolation method, a comparative study has supported the use of commercial kits such as miRCURY, ExoQuick, and Total Exosome Isolation Reagent as an adequate alternative to ultracentrifugation [35]. This study showed that all isolation techniques produced EVs within the expected size range (40–150 nm) and similar physical properties.

Transmission electron microscopy (TEM) imaging was performed on h-Islet-EV samples re-suspended in TBS, revealing a diverse population of particles (Fig 1D). EVs below 50 nm are observed in Fig 1D, contrasting with the NTA data where few particles less than 50 nm were measured. Differences in EV size as determined by NTA and electron microscopy has previously been described in other studies [36]. These differences could be attributed to the sample preparation process for electron microscopy, and the detection limitations of nanoparticle tracking analysis whereby EVs between 20–60 nm as observed by electron microscopy were not measured by NTA [37]. Nevertheless, the resuspension in TBS, required for TEM, did not alter the size distribution profile of the EVs as measured by NTA (Figure B in S1 File).

### h-Islet-EVs express EV markers

**Fig 2:**
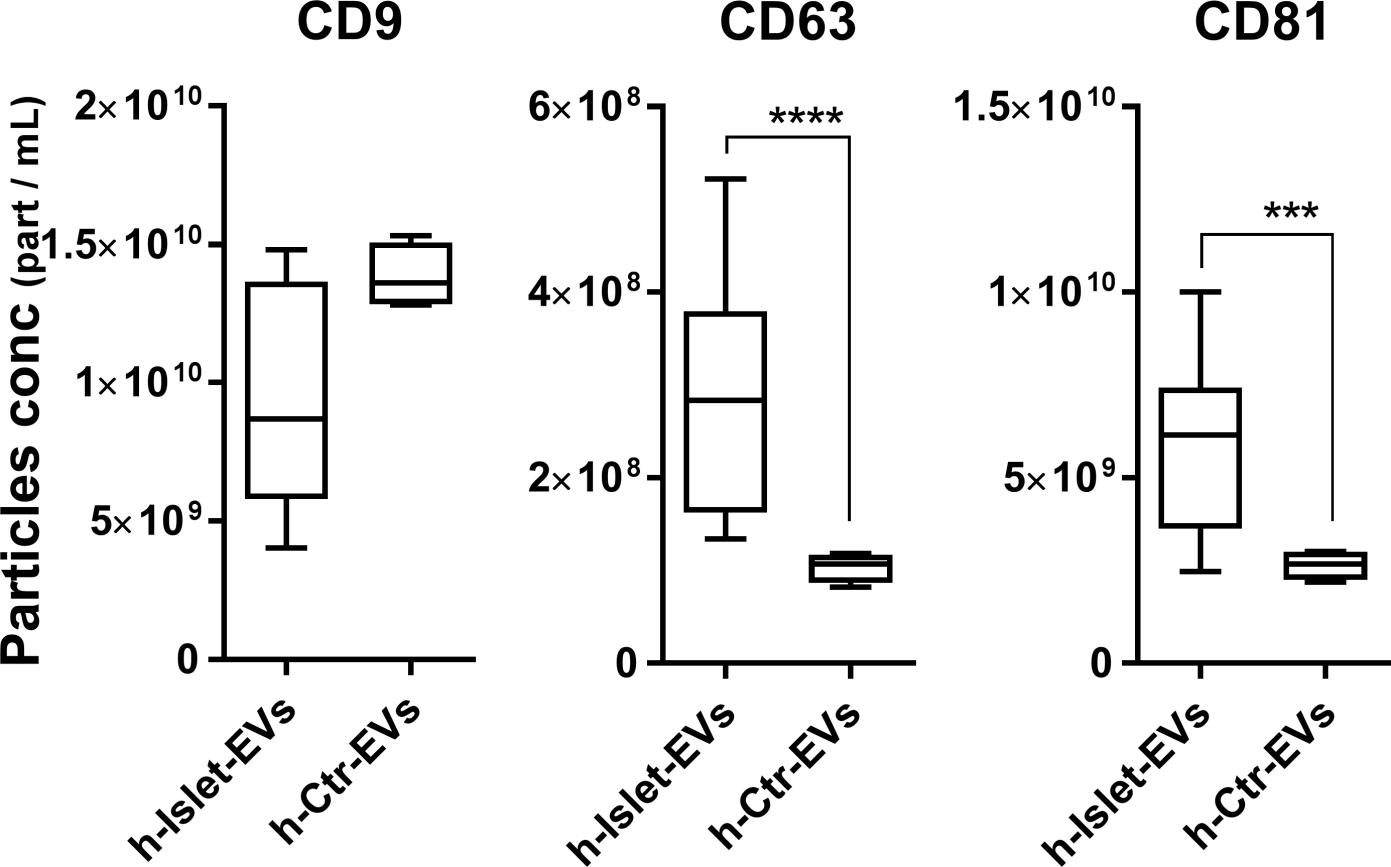
Quantification of CD63, CD9 and CD81 content in EVs by ELISA. Normalization was performed to the EVs protein content (N = 5). * represent statistical significance, and correspondingt p-value description in Table C in S1 File.

Tetraspanins are present in the membrane of EVs and are often used as EV biomarkers [25, 38, 39]. Therefore h-islet-EVs were analyzed for the presence of tetraspanins CD9, CD63 and CD81 by ELISA (Fig 2). No differences were observed in CD9 expression. However, the levels of both CD63 and CD81 were significantly higher in h-Islet-EVs compared to h-Ctr-EVs suggesting the release of a distinct population of EVs from h-islet cells. Literature suggests that accumulation of a specific tetraspanin family marker in EVs might be cell origin-specific [40].

### h-Islet-EV pancreatic cargo

**Fig 3:**
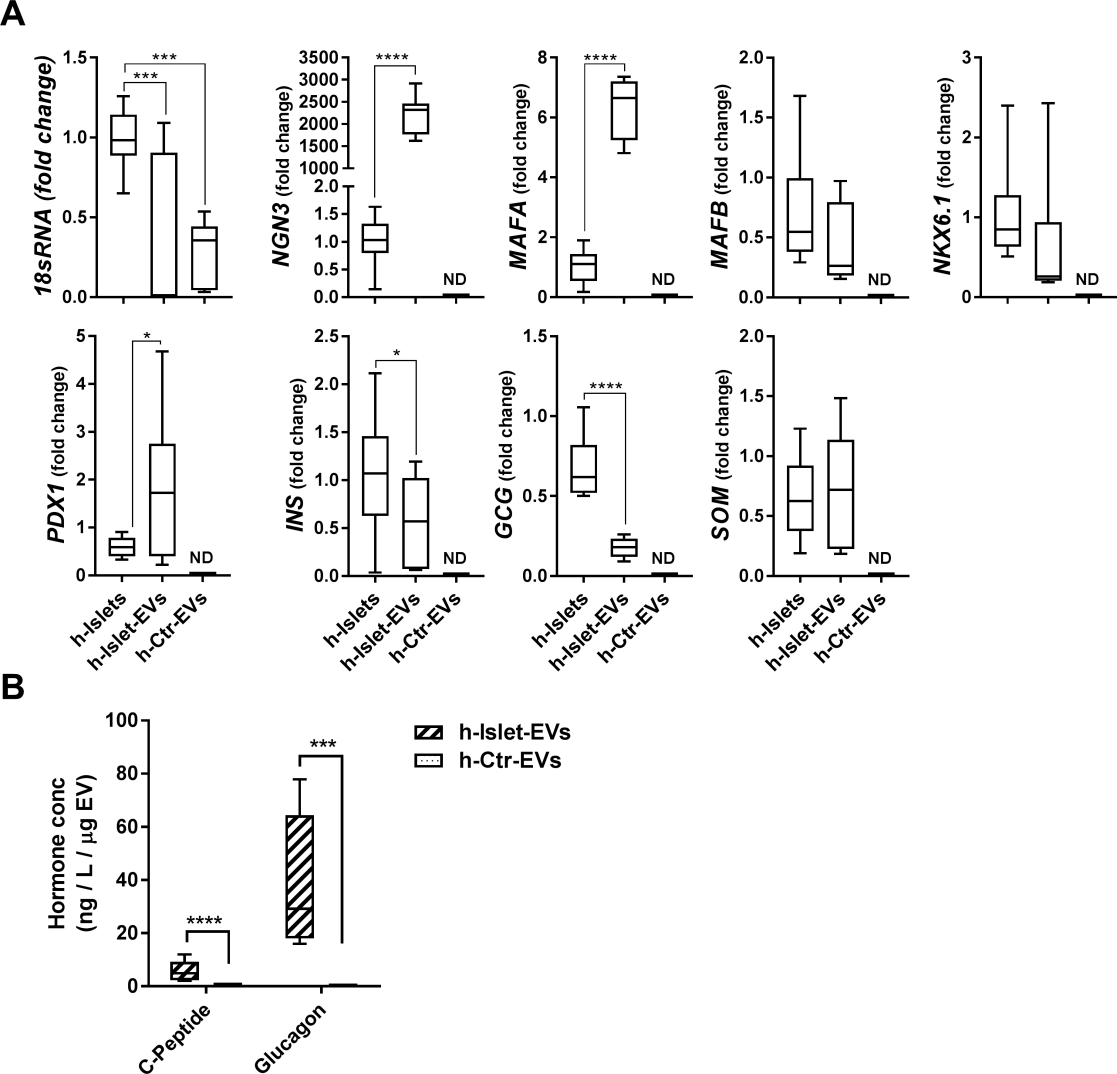
h-Islet-EV pancreatic cargo characterization. A) mRNA levels of pancreatic markers in h-Islet-EVs and h-Ctr-EVs as compared to h-islets. Fold change normalized against h-Islets (N = 3). B) Quantification of C-peptide and glucagon in h-Islet-EVs (N = 4) and h-Ctr-EVs (N = 3), normalized by EV protein content (μg). * represent statistical significance, and corresponding p-value description in Table C in S1 File.

The presence of protein and mRNA for pancreatic markers was analyzed in h-islet cells, h-islet-EVs and h-Ctr-EVs (Fig 3A). The housekeeping gene 18sRNA was used as a positive control. mRNA for 18sRNA was significantly higher in h-islet cells as compared to h-islet EVs or control-EVs, but no differences were observed between the EV populations. mRNA for pancreatic markers was not detected in control EVs. However, several pancreatic markers were found in h-islet-EVs. The presence of mRNA for pancreatic transcription factors, such as *PDX1*, *NGN3* and *MAFB* was significantly higher in h-Islet-EVs as compared to h-islets. *NKX6*.*1* and *MAFB* mRNA were detected in both h-islets and h-Islet-EVs, but no significant differences were observed. The mRNA for the hormone markers, *INS* and *GCG* was found significantly higher in h-Islets than in h-Islet-EVs, but no differences were observed in *SOM* mRNA expression. NGN3 is a critical transcriptional factor required for endocrine fate determination in the developing pancreas, and is thought to be absent postnatally in human islets [41, 42]. MafA and PDX1 are β-cell-specific and known to control glucose-responsive transcription of insulin and other genes in islet β-cells [43, 44]. The high concentration of both these mRNAs in the h-islet-EVs may suggest that pancreatic EVs may have a role in the endocrine fate specification via NGN3 signaling, and β-cell maturation and identity.

The protein levels of the two main pancreatic hormones found in h-islets, C-peptide and glucagon, was assessed by ELISA and compared between h-Islet-EVs and h-Ctr-EVs (Fig 3B). Significantly higher amounts of C-peptide and glucagon were detected in h-Islet-EVs, as compared to h-Ctr-EVs. These data reveal that h-Islet-EVs carry pancreatic genetic and protein information.

### Temporal commitment of iPSC clusters differentiated in suspension

2D-culture adaptation to 3D-suspension cluster culture involved culturing single iPSCs in baffled flasks in suspension and with rotation (Figure C (A) in S1 File). 3D-suspension culture led to a significant decrease in cell viability (Figure C (B) in S1 File). Even so, suspension culture viability remained above 85% during the suspension adaptation protocol. Cells within the iPSC cluster proliferated, which resulted in increased cell number and cluster diameter size over time (Figure C (C and D) in S1 File). The pluripotent maker OCT4, a typical embryonic/iPS stem cell marker [45], could be detected in iPSC clusters (Figure C (E) in S1 File). Dispersion of the iPSC clusters into single cells (Figure C (F) in S1 File) allowed a detailed visualization and quantification of OCT4 relative expression (Figure C (G) in S1 File). 89.5±6.7% (mean ±SD) of the cells stained positive for OCT4^+^ (normalized for Hoechst staining).

For iPSC cluster differentiation, a protocol adapted from Russ et al. [15] was used, and the iPSC cluster transcriptional profile is shown in Figure D in S1 File. Changes in mRNA expression over time were compared to undifferentiated iPSC clusters (day 0). The expression of *OCT4* gene was reduced at early culture time points and reached very low levels by the end of the differentiation protocol. Combined action of activin A (ActA) and Wnt3 treatment induced a significant expression of typical endoderm markers *SOX17* and *FOXA2* genes [11]. Overall, the mRNA expression of critical pancreatic transcription factors such as *NGN3*, *NEUROD1* and *MAFB* [46, 47], increased significantly over time, as observed from day 10 to day 22. *NKX6*.*1* and PDX1 showed a significant increase in mRNA after day 10. Contrasting with the other pancreatic transcription factors, *MAFA* gene expression was only significantly increased towards the end of the differentiation protocol (day 20–22). The expression of mRNA for pancreatic hormones was only observed at the latest time points of the differentiation. A significant increase in *SOM* expression was detected at day 10, followed by a peak in expression at day 16. *GCG* and *INS* mRNA expression reached significant levels by day 16 and onwards.

### EVs affect iPSC cluster differentiation in a dose-dependent fashion

EVs are well recognized for their ability to mediate cell-to-cell communication. In particular, MSC-derived EVs have been intensively investigated. In fact, EV mediated RNA delivery from hMSCs to human islets revealed an immunosuppressive effect capable of improving islet transplantation [48]. MSC-derived EVs are also known to mediate regenerative responses following hepatic injury [49]. Nevertheless, a growing interest is being devoted to tissue-derived EVs. Recent studies suggest that tissue homeostasis can be regulated via EV communication [50, 51].

**Fig 4:**
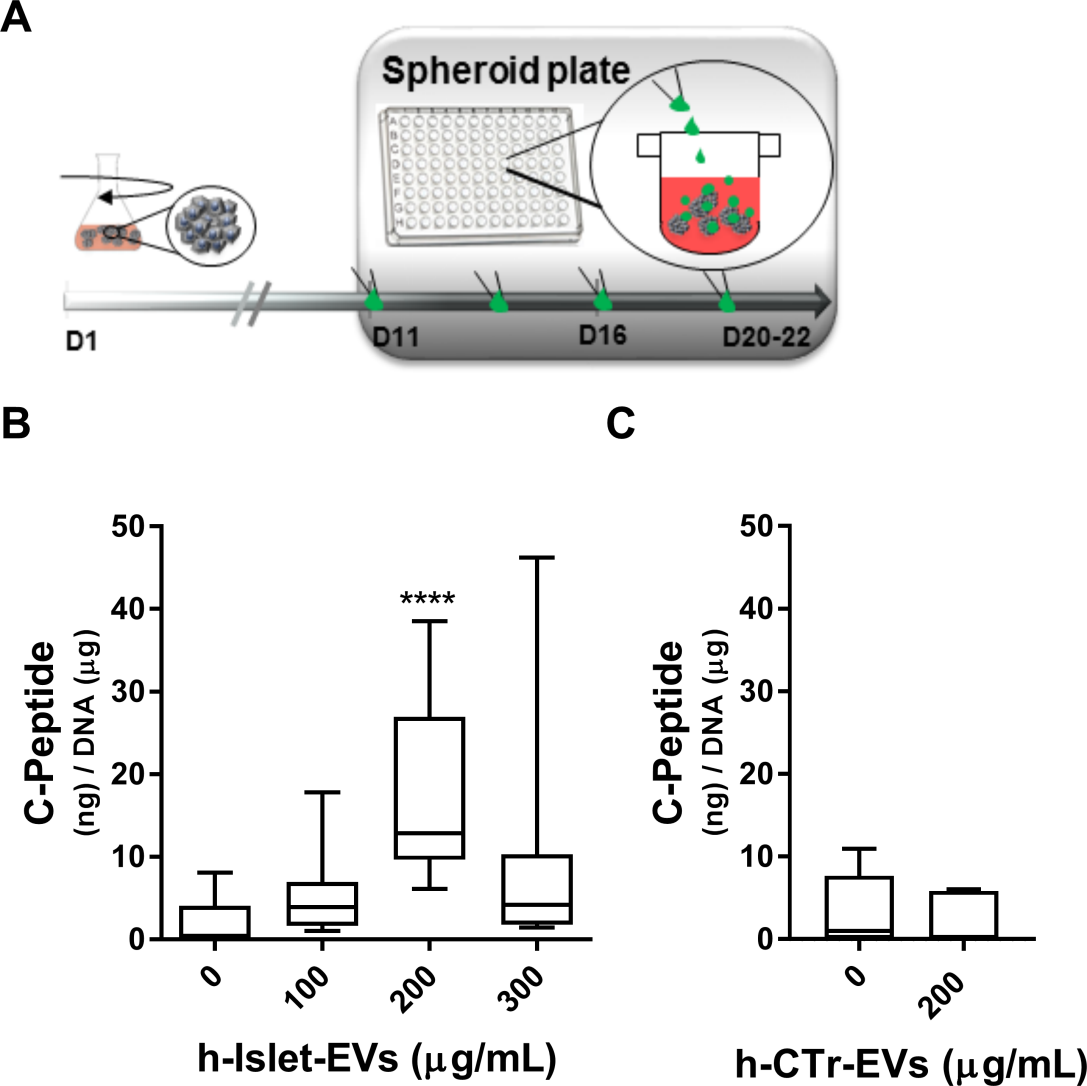
Dosage effect of h-islet-EV supplementation in iPSC clusters differentiated in suspension. A) Scheme of the timing of EVs supplementation. B) Quantification of C-peptide in iPSC clusters supplemented with increasing concentrations of h-Islet-EVs (N = 4). C) Quantification of C-Peptide in iPSC clusters supplemented with 200 μg/mL of h-Ctr-EVs. ELISA quantification was normalized by total DNA content. * represent statistical significance as compared with 0 μg/mL condition. Corresponding p-value description in Table C in S1 File.

To assess the effect of h-islet-EVs on stem cell differentiation, iPSC clusters at day 11 of differentiation were transferred to spheroid 96 well plates. At this stage of differentiation the iPSC clusters had a mean diameter size of 189.4±36 μm (Figure E in S1 File). Increasing concentrations of h-Islet-EVs (0, 100, 200 and 300 μg/mL) were added to the iPSC clusters at each media change from day 11 until 20–22 (Fig 4A). Supplementing iPSC clusters with 300 μg/mL h-islet-EVs, significantly decreased mRNA expression of *INS* and *GCG* in the iPSCs (Figure F (A and B) in S1 File). *SOM* mRNA showed a significant increase in expression which correlated with increasing doses of h-islet-EVs (Figure F (C) in S1 File).

Aqueous cell lysates were used to measure the total intracellular C-peptide, and assess the EV supplementation effect at the protein level. Addition of 200 ug/mL h-Islet-EVs to iPS clusters led to a significant increase in the intracellular levels of C-peptide, and this effect was observed to be a concentration-dependent effect (Fig 4B). In this condition, an average of 17.5 ng of C-peptide per μg of DNA was quantified in the iPSC cluster lysates. Supplementation of 200 μg/mL of EVs (in 100 μL) may have contributed to an average accumulation of 0.11 ng of C-peptide. The increase in intracellular C-peptide levels may be due to EV-mediated signaling leading to increased protein expression and/or due to EV accumulation inside the iPSC clusters.

As a disclosure, even at the most favorable condition, in this work the intracellular levels of C-peptide in the iPS clusters were found approximately 100 times less as compared to the original work described by Russ et al. [15].

Supplementation of h-Ctr-EVs to iPS_clu_, at 200 μg/mL, did not induce changes in the intracellular levels of C-PEP protein (Fig 4C). H-Ctr-EVs contain approximately 2x10^6^ particles per μg of protein, while h-Islet-EVs contain approximately 9x10^5^ particles per μg of protein. In this work a protein dosage was used, and the effects of dosing in particle concentration was not explored. The dosing of EVs in particle concentration or mRNA concentration is of potential interest to fully understand the EV-mediated communication and regulatory process.

Nevertheless, this data is in accordance with literature suggesting that factors released by the developing pancreas may be instrumental in engineering stem cells [52]. Stem cells, in particular MSCs, have also been shown to be affected by other cell-derived EVs. Exosomes derived from neuron progenitor cells at various differentiation stages could differentiate hMSC into neuron-like cells, via delivery of miRNAs known to play a role in neuronal differentiation [53]. Similarly, EVs derived from a murine pancreatic β-cell line applied subcutaneously using Matrigel^TM^ platforms containing bone marrow cells, elicited a long-term control of glucose levels over 60 days in diabetic immunocompromised mice [54].

### EV delivery to iPSC clusters cultured in 3D-collagen hydrogels does not affect insulin expression

To address whether delivery of h-Islet-EVs to iPSCs in 3D-matrices could simultaneously support 3D-culture of stem cells while inducing differentiation by the release of the nano-cargo content, EVs were supplementated to iPSC clusters cultured in 3D-collagen. iPSC clusters were cultured in 3D-collagen hydrogels from day 7 of differentiation until day 16 (COL D16) and compared with iPSC clusters differentiated until day 21 in suspension (Susp D21), as illustrated in Figure G (A) in S1 File. 3D-culture in collagen hydrogels induced a significant increase in the mRNA expression of pancreatic transcription factors (Figure G (B) in S1 File) *NGN3*, *NKX6*.*1* and *PDX1*. mRNA expression of pancreatic hormones, *INS*, *GCG* and *SOM*, also increased upon 3D-culture in collagen hydrogels. However, only *INS* mRNA showed a significant fold change increase. Collagen hydrogels have been found suitable for 3D-culture and neuronal differentiation of iPS cells, with beneficial outcome in transplantation when used in an animal model [55]. Generally, hydrogels due to their unique biocompatibility, flexible methods of synthesis, range of constituents, and tunable physical characteristics, have been the material of choice for many applications in tissue engineering and regenerative medicine [56].

**Fig 5:**
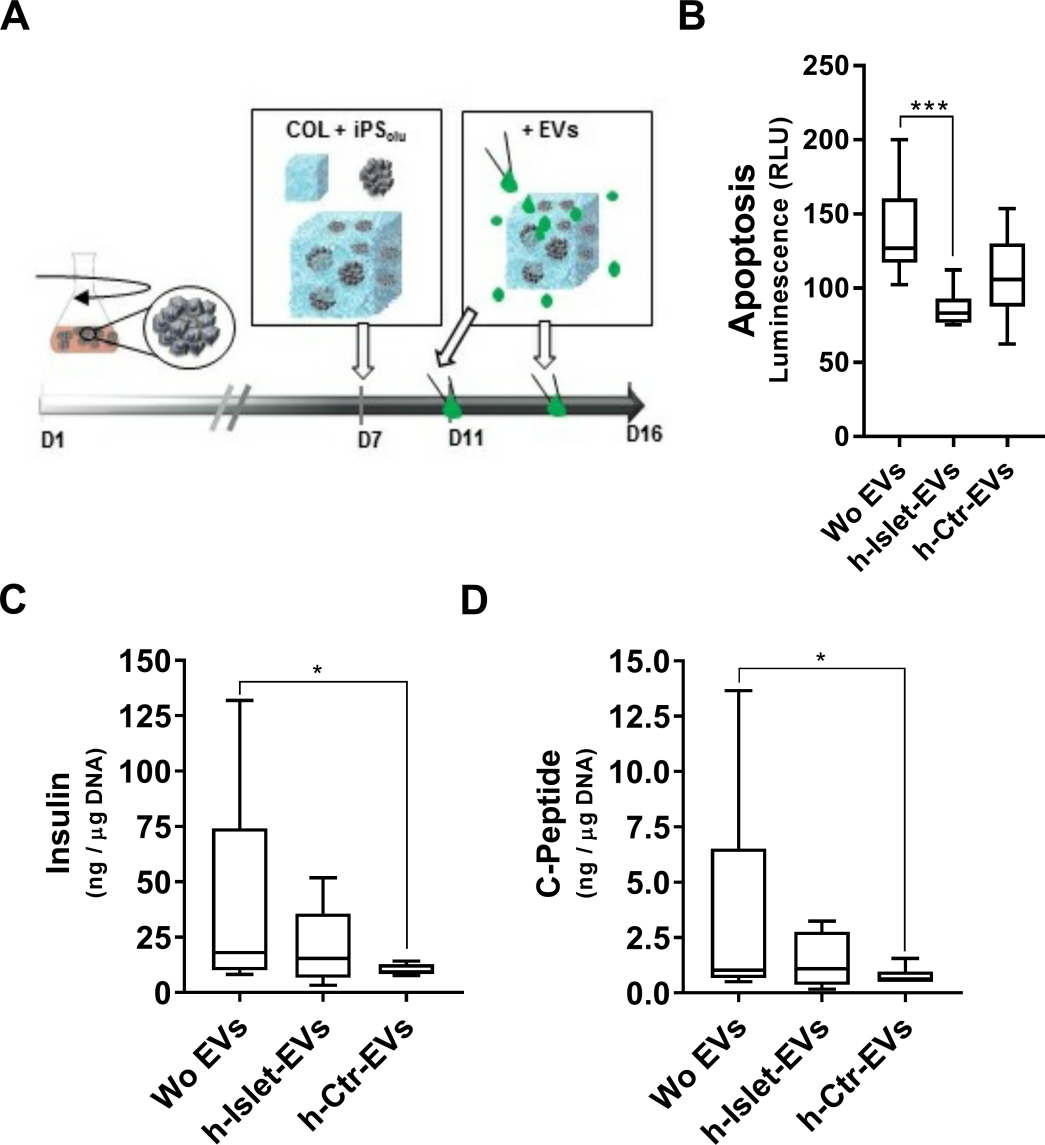
EV supplementation to differentiating iPSC clusters in 3D-COL hydrogels. A) Protocol scheme; B) Relative Luminescence Units (RLU) measured from the Apoptosis assay (N = 3). Quantification of intracellular C) insulin (N = 3) and D) C-peptide (N = 3) by ELISA, normalized to total dsDNA content. “WoEVs” denotes iPSC clusters cultured in 3D-COL without supplementation of EVs. * represent statistical significance. P-value description in Table C in S1 File.

EVs were added, in the differentiation media, to iPSC clusters cultured in 3D-collagen hydrogels (Fig 5A). H-islet-EV delivery to iPSC clusters led to significantly lower levels of apoptosis as compared to iPSC clusters cultured in 3D-hydrogels without EV stimuli (Fig 5B). This feature could be beneficial for the in-vitro culture of these constructs, when aiming for long-term assays and/or subsequent transplantation. Islet-derived EVs have been shown to protect β-cell endothelium from apoptosis when delivered in the culture media [31], endorsing the results described here. iPSC cluster treatment with h-Ctr-EVs reduced apoptosis levels as compared to no EV treatment condition, but no significant differences were observed.

Intracellular levels of insulin and C-peptide protein were measured (Fig 5C and 5D) but no significant changes were observed in 3D-culture with or without h-Islet-EVs, contrasting with the observations of EV delivery to iPSC clusters differentiating in suspension. However, a significant decrease in insulin and C-peptide levels was observed upon h-Ctr-EV supplementation, suggesting different regulatory mechanisms of the two EV populations. One must consider that human serum EVs may potentially carry a variety of signaling molecules and proteins such as albumin, transferrin, immunoglobulins, lipoproteins and others [57]. The composition of the h-Ctr-EVs was not explored in this work; however, the results described above may suggest the presence of an active cargo in the serum EVs that negatively regulate insulin expression in the differentiated iPSC clusters.

## Conclusion

This work aimed to characterize h-Islet-EVs and to investigate the effect of h-Islet-EVs on insulin expression in differentiating iPSC clusters. H-Islets released biologically active EVs that were able to induce a significant increase in iPSC clusters intracellular levels of C-peptide, in a concentration-dependent way. 3D-culture of iPSC clusters in collagen hydrogels supplemented with h-Islet-EVs did not affect insulin expression, however a regulation of cell apoptosis was observed.

The identified regulatory effects may be of significance for future pancreatic differentiation models. As a next step a detailed transcriptomic and proteomic analysis of the h-Islet-EVs may lead to the identification of novel molecular mechanisms underlying pancreatic regulation and commitment. Isolation of EVs from longer term cultures of h-islets may also bring more knowledge about the temporal release of active factors and association with apoptotic signaling, and how such signaling affects iPSCs differentiation and viability.

## Supporting information

S1 File Combined supporting information file contains Materials and methods A to G, Figures A to G, and Tables A to C.
(DOCX)

## Abbreviations

H-Islets: human pancreatic islets
iPSC: induced pluripotent stem cells
EVs: extracellular vesicles
h-Islet-EVs: human pancreatic islet-derived EVs
h-Ctr-EVs: human serum-derived EVs
