# Supplementary material for: Human pancreatic islet-derived extracellular vesicles modulate insulin expression in 3D-differentiating iPSC clusters
Source: PLoS One. 2017 Nov 8;12(11):e0187665. doi: 10.1371/journal.pone.0187665 (PMC5678888; doi:10.1371/journal.pone.0187665)
Supplement: S1 File — Combined supporting information file contains Materials and methods A to G, Figures A to G, and Tables A to C. (DOCX) [file pone.0187665.s001.docx]

**Combined supporting information file (File S1)**

**Human pancreatic islet-derived extracellular vesicles modulate insulin expression in 3D-differentiating iPSC clusters**

Diana Ribeiro^†,§ *^, Eva-Marie Andersson^‡^, Nikki Heath^+^, Anette Persson-kry^§^, Richard Collins^⊥^, Ryan Hicks^§^, Niek Dekker^§^, Anna Forslöw^§*^

**Content**

Materials and methods A to G

Figures A to G

Tables A to C

**Supplementary materials and methods**

- 1. **Materials and media**

PRODO ISLET MEDIA (PIM)® was purchased from Prodo Laboratories, Inc. DEF-CS™ media with supplements (#Y30010) was purchased from Cellartis® Takara. DMEM (#SH30081.01) was purchased from HyClone. RPMI 1640 Medium, GlutaMAX™ Supplement (# 618700) was purchased from ThermoFisher Scientific^TM^. CMRL media (#11530-037), Heat inactivated FBS (#10270) and penicillin-streptomycin (#15140) were purchased from Invitrogen. Activin A (#120-14E) was purchased from Peprotech. WNT3a (#1324-WN), EGF (#236-EG-01M), and FGF7 (#251-KG) were purchased from R&D Systems. [ROCK Inhibitor Y-27632](https://www.stemcell.com/y-27632.html) (#688000) and TGFβi IV (#616454) were purchased from Merck Millipore. ALKi 5 Inhibitor II (#alx-270-445) was purchased from Enzo Life Sciences. LDN193189 (#04-0074) was purchased from Stemgent. Laminin 511^TM^ (#LN-511) was purchased from BioLamina. HEPES buffer (#15630), B27 (#0080085-SA), GlutaMAX^TM^ (#10565), MEM NEAA (#11140), ITS (#41400), and PBS (#10010-023) were purchased from Gibco. Tryplan Blue (#93595), TTNBP (#71441-28-6), Collagen from rat tail (#7661), Acetic acid (#64-19-7), NaHCO_3_ (#S6014), Triton (#T9284) were purchased from Sigma-Aldrich. Actin (#A12379), and Hoechst (#33342) were purchased from Molecular Probes^TM^. ExoQuick-TC^TM^, ExoCET exosome quantification assay kit, ExoElisa for CD63, CD9 and CD81 were purchased from Systems Biosciences. Pierce™ BCA Protein Assay Kit (#23227), Quant-iT™ PicoGreen ® dsDNA (#P7589) and TRIzol® Reagent (#15596026) were purchased from ThermoFisher Scientific^TM^. Caspase Assay was purchased from Promega. Mercodia Ultrasensitive Insulin ELISA (#10-1132-01), Mercodia Ultrasensitive C-peptide ELISA (#10-1141-01) and Mercodia Glucagon ELISA (#10-1271-01) were purchased from Mercodia. RNeasy® Mini Kit (#74106) was purchased from Qiagen. High-Capacity cDNA Reverse Transcription Kit (#4368813), TaqMan® Gene Expression Master Mix (#4369016), and MicroAmp® Optical 384-Well Reaction Plates (#4309849) were purchased from Applied Biosystems^TM^. 96 well ultra-low attachment multiwell spheroid plates (#4520) and Corning® 125 mL baffled Erlenmeyer flasks (#431405) were purchased from Corning®. 96 well glass bottom plates (#P96-1.5H-N) were purchased from In vitro Scientific.

- 1. **Gene expression analysis**

RNA was extracted using the RNeasy® Mini Kit following manufacturers’ instructions. RNA concentration for each sample was determined using a Nanodrop instrument (NanoDrop, Saveen Werner) then diluted to a concentration of 20 ng/μL. RNA was converted to cDNA by reverse transcription according to manufacturers’ instructions, using a High-Capacity cDNA Reverse Transcription Kit and Bio-Rad thermal cycler (C1000 Touch, Bio-Rad). Quantitative real-time PCR (qPCR) was performed by mixing 2 µL cDNA (4 ng/ μL) with 2.5 µL of RNase free water, 5 µL of TaqMan® Gene Expression Master Mix and 2 µL TaqMan® gene expression primers (S2 Table), to a final volume of 10 µL. MicroAmp® Optical 384-Well Reaction Plates were used and qPCR assays were performed in standard layouts for comparative Ct TaqMan®, in accordance with QuantStudio 7 Flex software and instrument instructions (Applied Biosystems^TM^).

Data was normalized to the housekeeping gene Glyceraldehyde-3-phosphate-dehygrogenase (GAPDH) and quantification performed using the ΔΔCT method. Cell clusters at specific time points were used as the calibrator sample (threshold = 1). In the absence of qPCR product signal, for the calibration sample, the Ct was set as 40 (maximum number of amplification cycles).

- 1. **Induced Pluripotent Stem (iPS) cells clustering**

A fibroblast-derived iPS cell line, described previously^1^, was expanded in the DEF-CS™ Culture System. iPS cell clustering was performed by adding 1.2x10^7^ cells per 40 mL of DEF-CS™ media in Corning® 125 mL Erlernmeyer flasks. In addition to the DEF supplements (GF1, GF2 and GF3), 10 µM Rock inhibitor was added to the DEF media. The cell suspension flasks were placed on an orbital shaker at 65 rpm to induce sphere formation for 72 hours. Afterwards, media was changed every day for the next 2 days, using DEF media supplemented with only GF1 and GF2. Cell viability and cell density was measured on a CedexHiRes instrument, using Tryplan Blue to identify non-viable cells (Ninolab Company).

- 1. **Pancreatic differentiation protocol**

The differentiation protocol used was adapted from Russ et al.^2^ Briefly, media – 1 (day 1): RPMI, 0.2% FBS, 1% penicillin-streptomycin, 1:5,000 ITS, 100 ng/mL Activin A, 50 ng/mL WNT3a and 10 µM Rock inhibitor. Media – 2 (day 2 and 3), comprising RPMI, 0.2% FBS, 1% penicillin-streptomycin, 1:2,000 ITS, and 100 ng/mL Activin A. Media - 3 (day 4): RPMI, 0.2% FBS, 1% penicillin-streptomycin, 1:1,000 ITS, 25 μM TGFβi IV and 25 ng/mL FGF7. Media – 4 (day 5 and 6); RPMI, 0.4% FBS, 1% penicillin-streptomycin, 1:1,000 ITS and 25 ng/mL FGF7; Media - 5 (day 7 and 8): DMEM, 1% penicillin-streptomycin, 1:100 B27 and 3 nM TTNBP; Media - 6 (day 9): DMEM, 1% penicillin-streptomycin, 1:100 B27, 3 nM TTNBP and 50 ng/mL EGF; Media - 7 (day 10): DMEM, 1% penicillin-streptomycin, 1:100 B27, 50 ng/mL EGF and 50 ng/mL FGF7. Media - 8 (day 11-15): DMEM, 1% penicillin-streptomycin, 1:100 B27, 500 nM LDN193189, 1 mM ALKi II and 25 ng/mL FGF7. Media - 9 (day 16-21): CMRL, 1% penicillin-streptomycin, 1:100 GlutaMAX and 1:100 NEAAs. Until Media - 8, medium change was performed daily, from this stage and fowards media change was performed every second day.

- 1. **Collagen hydrogels**

Collagen from rat tail was dissolved overnight at 4ºC to a stock concentration of 4 mg/mL, using 0.2 N acetic acid. Collagen hydrogels were produced on ice by neutralizing collagen with equal parts of 1M HEPES buffer and 37 g/L NaHCO_3_ to a final concentration of 1.5 mg/mL. The collagen solution was left in an ice-bath until used for cellular and/or EV encapsulation. 300 µL tips were used to pipette 15 µL gel mix (or gel mix+clusters) into 96 well glass bottom plates. Gels or gel+cluster mixes were left to jellify for 1.5 h at 37ºC, before adding cell culture media.

- 1. **Immunofluorescence protocol**

Suspension cell clusters were fixed in 3.7% formalin and stored at 4ºC in PBS until use. Samples were incubated with permeabilization / wash buffer (1% FBS+1% Triton in PBS) for 15 - 20 minutes, followed by a PBS wash. Blocking buffer, composed of 1% BSA in PBS, was added to the samples and incubated for 1h. Afterwards, the samples were incubated overnight at 4ºC in diluted primary OCT3/3 rabbit poplyclonal antibody (Cell Sig. Technology, #2750, dilution 1:400) prepared with permeabilization / wash buffer. Samples were rinsed in PBS, following 5h incubation with the diluted secondary antibody solution (Alexa Fluor 594, Life Technologies, #A11037, dilution 1:650) prepared with permeabilization / wash buffer. Controls were performed by adding permeabilization / wash buffer without primary antibody, while keeping the secondary antibody incubation. Finally, samples were incubated with Hoechst (dilution 1:2000) for 15 min, for nuclear staining. Stained samples were stored in PBS for no longer than a week until imaging.

In parallel, suspension cell clusters were enzymatically dissociated to single cells and seeded in laminin 511 (5 µg/µL) coating and cultured for additional 2 to 3 days. The dissociated cells were afterwards fixed and labelled as described before, however using a permeabilization / wash buffer composed of 1% FBS and 0.1% Triton in PBS. These samples were used for the imaging quantification of the stains.

A Nikon Confocal Laser Scanning Microscope System, composed of an inverted Eclipse TE2000-E microscope, was used for confocal imaging. Image acquisition was performed using EZ-C1 Software (Nikon Corporation).

- 1. **Imaging analysis**

All image analysis was performed using FIJI – Image J software (open source).

Fluorescence-based images were adjusted to the same gain by adjusting the laser intensity of exposure to the sample with highest signal, then using that gain to normalize all the samples for each specific marker. Control secondary antibody only staining was used to subtract cell auto-fluorescence and background staining. A threshold-based analysis was defined for automatic selection of the cell cluster area or nuclei. Quantification of the expression profile was measured by normalizing labelled protein intensity /intensity of nuclear staining, and using control secondary antibody as a staining threshold. Morphometric analysis was performed by measuring the diameter size of the clusters.

References

1. Sjogren AK; Liljevald M; Glinghammar B; Sagemark J; Li XQ; Jonebring A; Cotgreave I; Brolén G; TB, A., Critical differences in toxicity mechanisms in induced pluripotent stem cell-derived hepatocytes, hepatic cell lines and primary hepatocytes. *Arch Toxicol.* **2014,** *88* (7), 1427-37.

2. Russ, H. A.; Parent, A. V.; Ringler, J. J.; Hennings, T. G.; Nair, G. G.; Shveygert, M.; Guo, T.; Puri, S.; Haataja, L.; Cirulli, V.; Blelloch, R.; Szot, G. L.; Arvan, P.; Hebrok, M., Controlled induction of human pancreatic progenitors produces functional beta-like cells in vitro. *The EMBO Journal* **2015,** *34* (13), 1759-72.

**Supplementary figures**

**Figure A**

|  |
| --- |
| **Figure A**. **h-Islet-EV and h-Ctr-EV protein concentration per particle.** |

**Figure B**

| **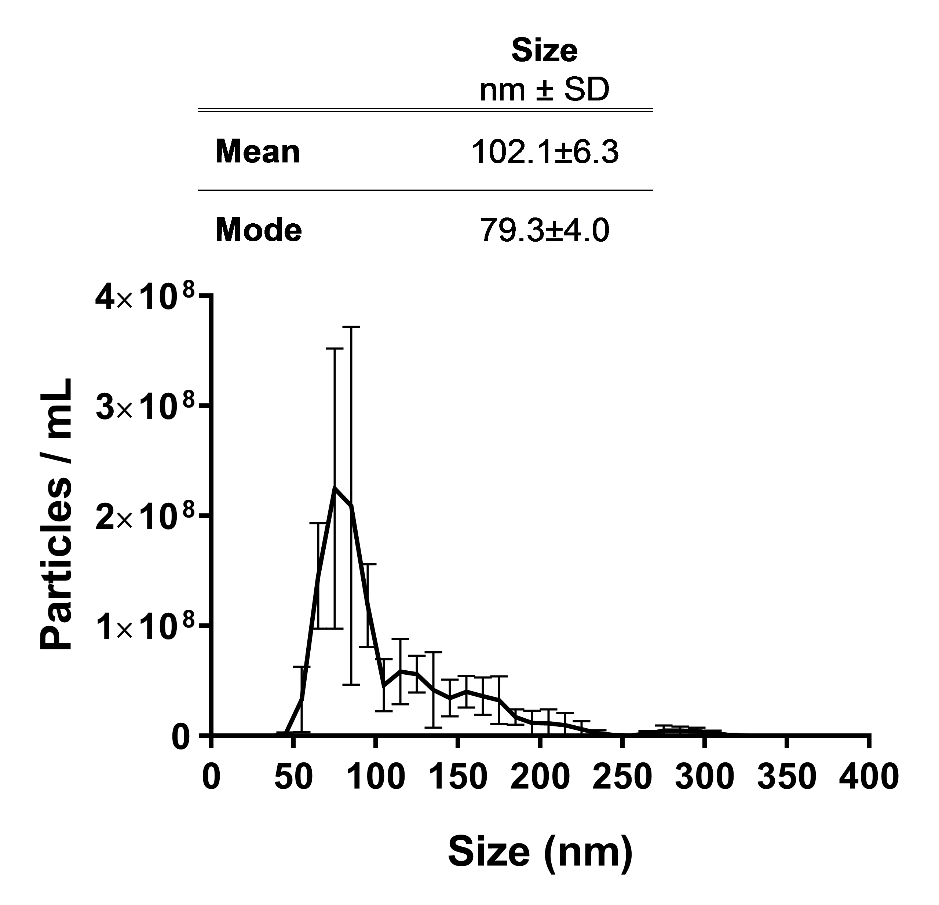** |
| --- |
| **Figure B**. **h-Islet-EV size distribution, in TBS buffer.** Size distribution over frequency of events. Descriptive statistics listed in table above the graph. |

| **Figure C**  **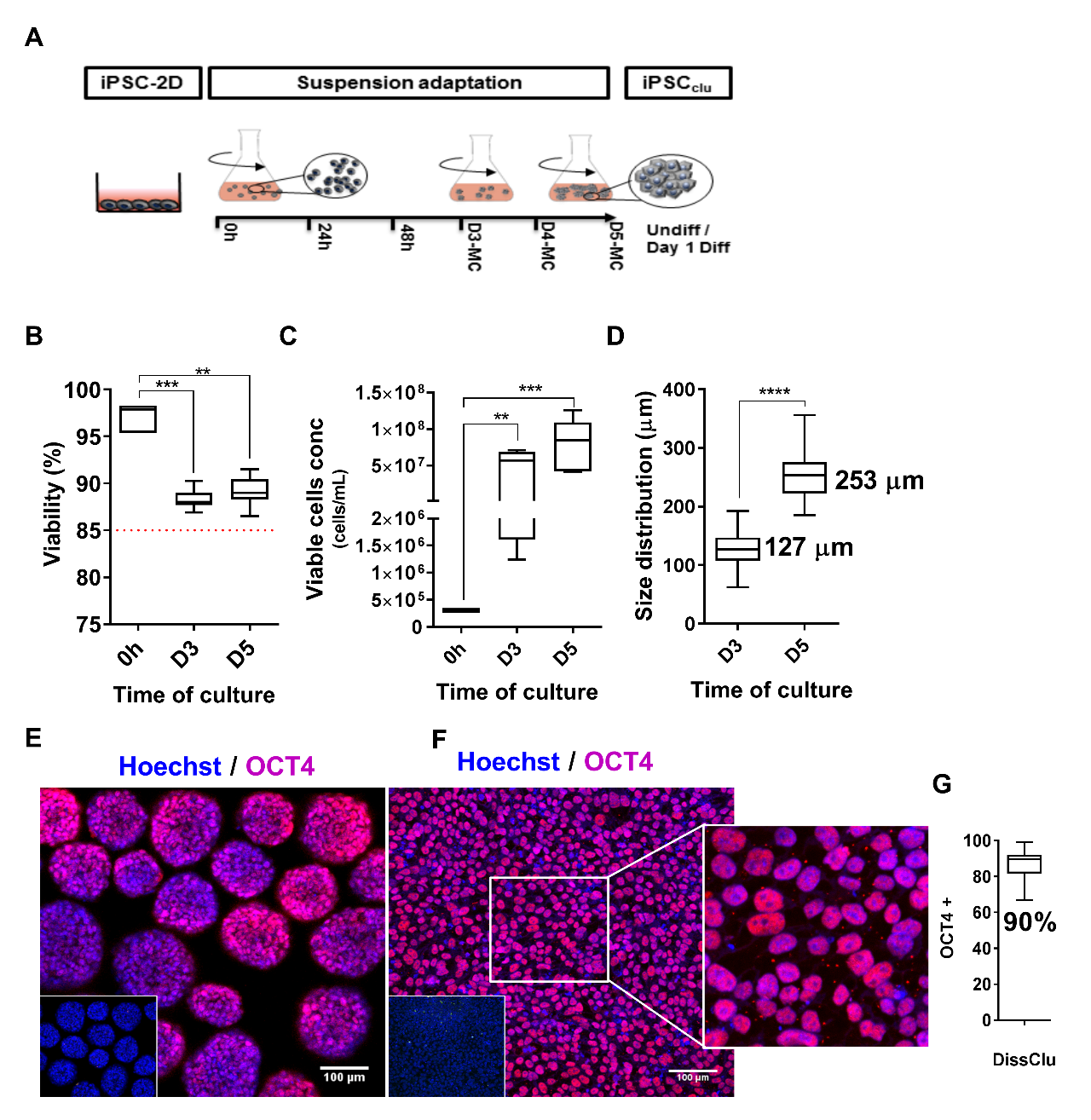** |
| --- |
| **Figure C**. **Characterization of iPSC clusters adapted to suspension culture.** A) Scheme of suspension culture adaptation protocol. “MC” media change; “Undiff” undifferentiated state; “Diff” differentiation; “Clu” clusters. B) Viability over time of culture. C) Viable cells concentration over time of culture. D) Cluster diameter size measurements at 72 hours and day 5. Average size value for each day stated next to the bar plot. E) Antibody staining of OCT4 in iPSC_clu_; Lower left panel – negative stain control image. F) Antibody staining of OCT4 in single cells dissociated from iPSC_clu_; Lower left panel – stain control imaging. G) Quantification of ratio of OCT4+ cells normalized by the nuclear expression of Hoechst. * represent statistical significance, and correspondent p-value description is on table S3. |

**Figure D**

| **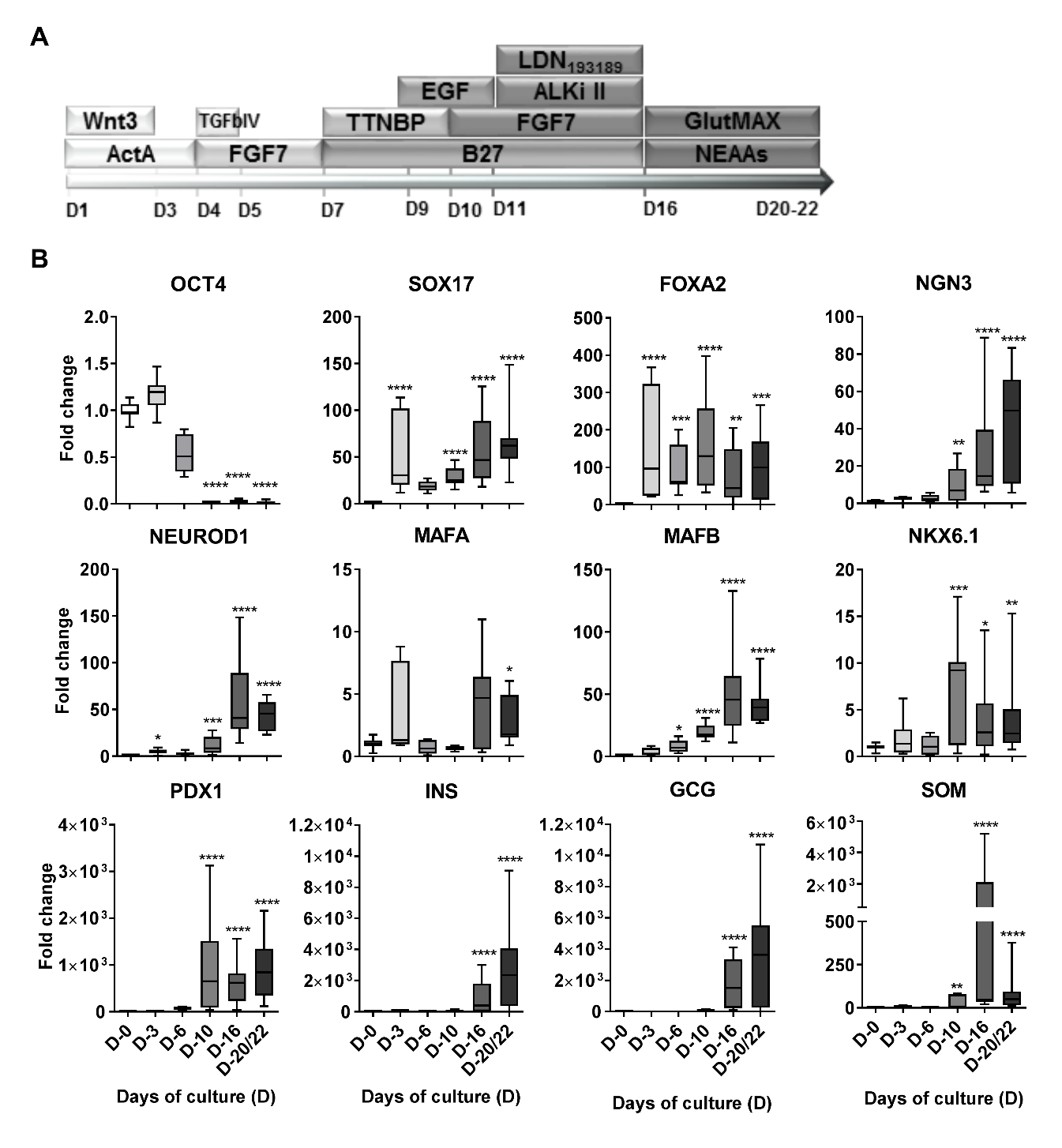** |
| --- |
| **Figure D.** **Characterization of iPSC_clu_ pancreatic differentiation profile.** A) Scheme of differentiation protocol, highlighting the timing and major differentiation factors. B) Gene expression over time. * represent statistical significance compared to day 0 of differentiation, and corresponding p-value description is on table S3. |

**Figure E**

| **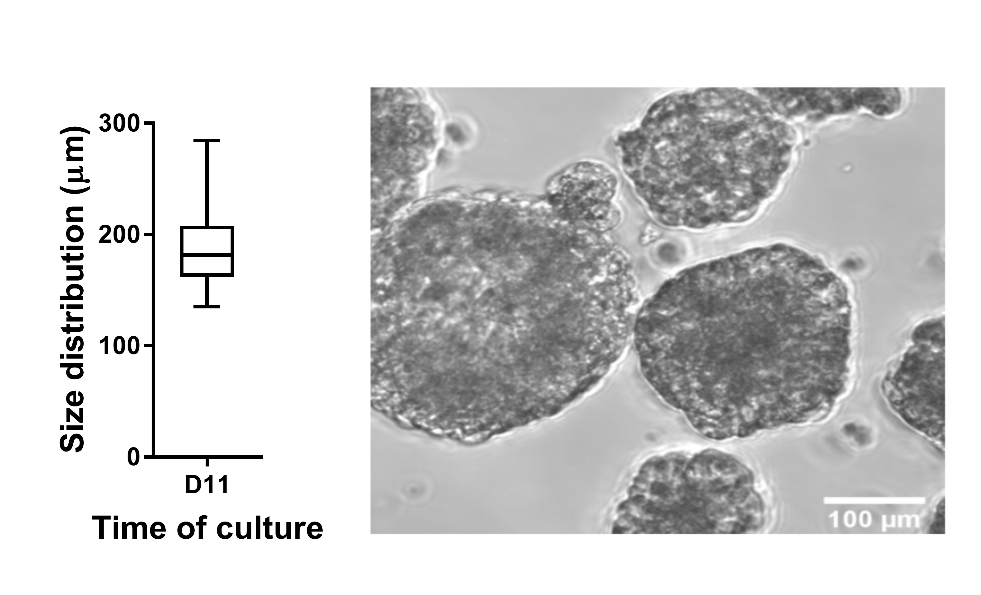** |
| --- |
| **Figure E.** **Morphology of iPSC_clu_.** Cluster diameter size distribution and contrast-phase images of iPSC clusters at day 11 of differentiation before EV supplementation in suspension. |

**Figure F**

| **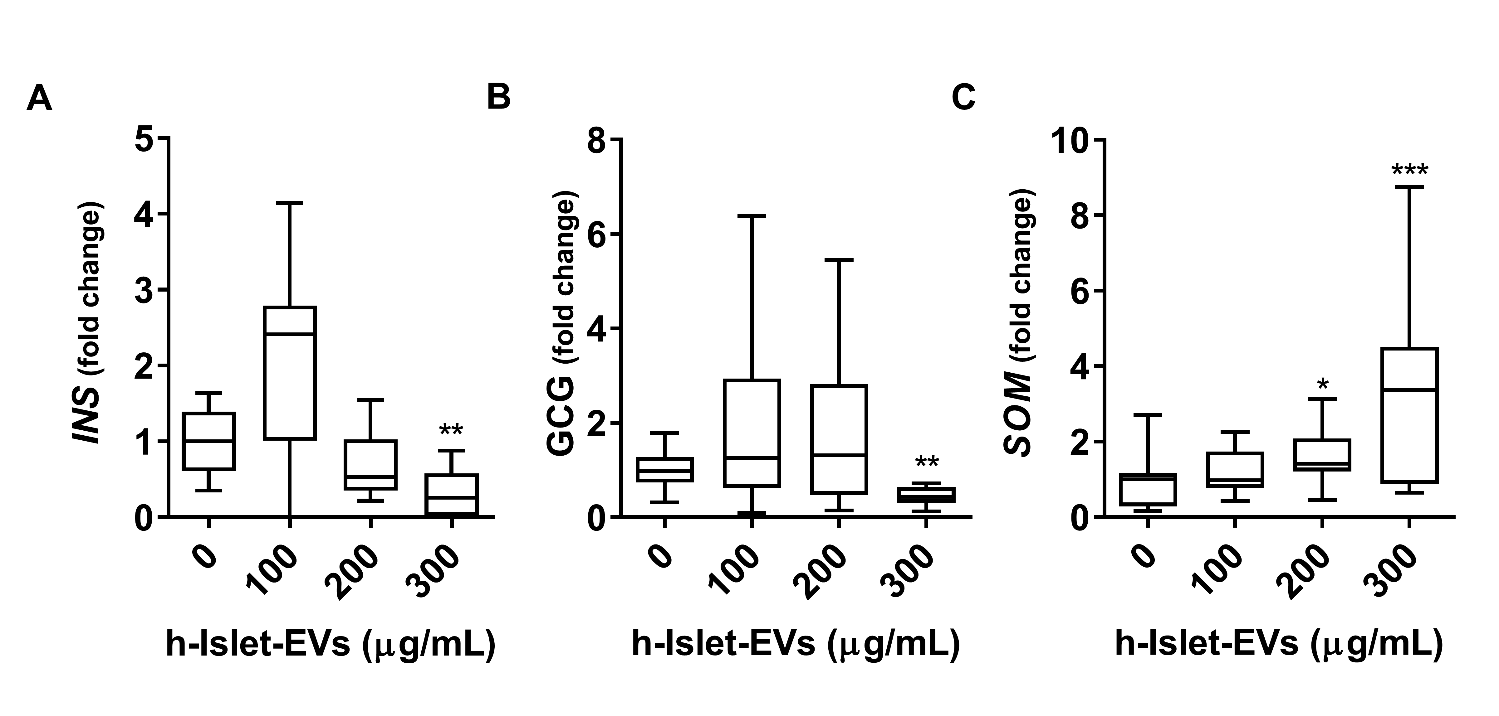** |
| --- |
| **Figure F**. **Dosage effect of h-Islet-EV supplementation to iPSC clusters differentiated in suspension.** A) *INS*, B) *GCG* and C) *SOM* mRNA expression. Fold change normalized against concentration 0 µg/mL. * represent statistical significance compared to 0 µg/mL condition, and correspondent p-value description is on table S3. |

**Figure G**

| **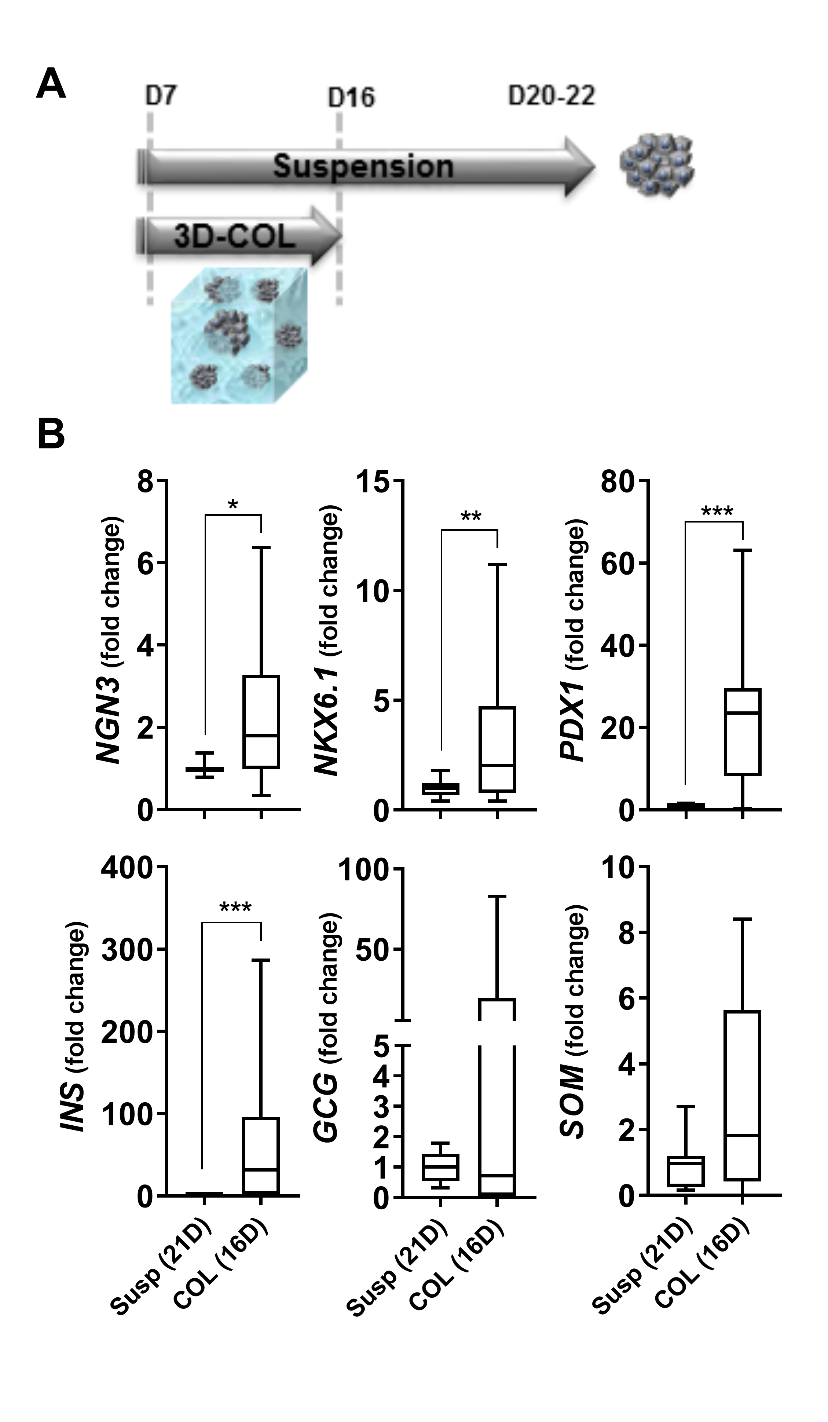** |
| --- |
| **Figure G**. **iPSC clusters pancreatic commitment in 3D-collagen culture.** A) Scheme of iPSC clusters embedded in the collagen hydrogels. B) Gene expression profile (N=3). Fold change normalized against iPSC clusters differentiated in suspension for 21 days (Susp). * represent statistical significance, and correspondent p-value description on table S3. |

**Supplementary tables**

**Table A. Information of the h-Islets donors’ samples used for EVs isolation.**

| **#** | **Gender** | **Age** | **BMI** | **Cause of Death** | **Diabetes history** |
| --- | --- | --- | --- | --- | --- |
| 1 | Male | 23 | 24,8 | Anoxia event | No |
| 2 | Male | 27 | 20,8 | Stroke | No |
| 3 | Male | 29 | 22,4 | Head trauma | No |
| 4 | Male | 32 | 25,1 | Anoxia event | No |
| 5 | Male | 41 | 28,4 | Anoxia event | No |
| 6 | Male | 53 | 33,5 | CVA/stroke | No |
| 7 | Female | 37 | 25,1 | Anoxia event | No |
| 8 | Female | 39 | 29,5 | Stroke | No |
| 9 | Female | 44 | 32,0 | Stroke | No |
| 10 | Female | 53 | 32,0 | Stroke | No |
| 11 | Female | 69 | 22,7 | Stroke | No |
|  | **(Avg±SD)** | **41±13** | **27±4** |  |  |

**Table B. List of TaqMan® gene expression assays, by Applied Biosystems^TM^**

| **Gene** | **Assay ID** | **Amplicon size (bp)** | **Reporter dye** |
| --- | --- | --- | --- |
| GAPDH | Hs02758991_g1 | 93 | FAM |
| OCT4 | Hs00999632_g1 | 77 | FAM |
| SOX17 | Hs00751752_s1 | 149 | FAM |
| FOXA2 | Hs00232764_m1 | 66 | FAM |
| PDX1 | Hs00236830_m1 | 73 | FAM |
| NKX6.1 | Hs00232355_m1 | 93 | FAM |
| NEUROD1 | Hs01922995_s1 | 110 | FAM |
| NGN3 | Hs01875204_s1 | 127 | FAM |
| MAFA | Hs01651425_s1 | 121 | FAM |
| MAFB | Hs00534343_s1 | 86 | FAM |
| INS | Hs02741908_m1 | 139 | FAM |
| GCG | Hs01031536_m1 | 86 | FAM |
| SOM | Hs00356144_m1 | 86 | FAM |
| COL1 | Hs00164004_m1 | 66 | FAM |
| COL3 | Hs00943809_m1 | 65 | FAM |
| COL4 | Hs00178621_m1 | 112 | FAM |
| COL5 | Hs00609133_m1 | 75 | FAM |

**Table C. P-value description.**

| **P-value** | **Wording** | **Symbol summary** |
| --- | --- | --- |
| < 0.0001 | Extremely significant | **** |
| 0.0001 to 0.001 | Extremely significant | *** |
| 0.001 to 0.01 | Very significant | ** |
| 0.01 to 0.05 | Significant | * |
| ≥ 0.05 | Not significant |  |
